# Health communication for AMR behaviour change: Zimbabwean students’ relationships with the microbial world

**DOI:** 10.1093/jacamr/dlad133

**Published:** 2023-12-11

**Authors:** Martin Mickelsson, Tecklah Usai, Dorothy Chinofunga, Emma Oljans

**Affiliations:** Department of Women’s and Children’s Health, Uppsala University, Uppsala University Hospital SE-751 85 Uppsala, Sweden; Faculty of Education, Department of Science Technology and Design Education, Midlands State University, Gweru, Zimbabwe; Faculty of Education, Department of Science Technology and Design Education, Midlands State University, Gweru, Zimbabwe; Department of Women’s and Children’s Health, Uppsala University, Uppsala University Hospital SE-751 85 Uppsala, Sweden; The Department of Movement, Culture and Society, The Swedish School of Sport and Health Sciences, Stockholm, Sweden

## Abstract

**Background:**

Microbes have a global impact on health; microbial relationships benefit and impair quality of life. Negative health impacts of antimicrobial resistance (AMR) in our relationships with the microbial world are primarily borne by the Global South

**Objectives:**

To study experiences, understandings and practices of Zimbabwean students regarding health, food and microbes.

**Methods:**

Using purposive sampling, Zimbabwean school students were recruited as participants in group interviews supported by participant observation, exploring the relationships between health, food and microbes.

**Results:**

The study included 120 students from six upper secondary schools in the Midland Region and Gweru District. Findings identify two categories: microbial relationships and microbial encounters, each with three subcategories. Food emerged as both mediating artefacts and mediating experiences, enabling the students to link biomedical explanations of AMR and their everyday lives with friends and family. The necessity for health communication to explore and engage with participants’ contextual preferences and motivations is highlighted. When discussing food choices and practices, students considered the beneficial relationships with the microbial world.

**Conclusions:**

A contextually relevant approach is outlined, where food mediates the relationship between student health and the microbial world, supporting health communication for AMR behaviour change. Expanding AMR education to include the everyday experiences of food enables students to link the pressing sustainability challenge of AMR to their health goals. The study showcases how the exploration of microbial relationships and food practices as a ubiquitous feature of community life can form a basis for AMR prevention and control.

## Introduction

This paper aims to explore experiences, understandings and practices of Zimbabwean students regarding health, food and microbes, contributing to health communication for antimicrobial resistance (AMR) behaviour change. Through a series of group interviews, the paper studies students’ discussions about food practices, the relationship between health and the microbial world, and ways of coexisting with microbes. The paper joins previous research moving beyond the ‘war metaphor’ in thinking about our microbial relationships, seeing microbes as potentially both beneficial and detrimental to human health.^1–3^ AMR includes challenges of overprescription, overuse and misuse of antimicrobials. The WHO^4^ has identified AMR as a global and national health challenge, requiring holistic approaches connecting human health to animal and environmental health.^5–8^ Education has, together with communication and information, emerged in WHO policy as key in addressing AMR.^4,9–13^ To this end, students need to learn about AMR and how it constitutes a risk to their health and the health of their communities.^14,15^ To extend education beyond reiterating biomedical knowledge^16–20^ and to provide contextual relevance, knowledge needs to be grounded in the experiences of students.^21^ While microbes are difficult to observe directly, students experience their effects positively and negatively.^22^ The paper contributes with insights on how students’ relationships with the microbial world can inform educational efforts to address the sustainability challenge of AMR.

## Materials and methods

Study conceptualization and preparation of the interview guide and observation schema were done with input from both the Swedish and Zimbabwean teams, with the latter further enhancing the contextual relevance of the methods used. A pilot study was conducted ahead of the group interviews, in which the interview guide was piloted for comprehension and understanding by the participants. The interview guide was subsequently adapted for enhanced linguistic accessibility. Data collection was planned to be conducted together on site. Design of the interview guide and observation protocol was guided by the research objective to study experiences, understandings and practices of Zimbabwean students regarding health, food and microbes. Due to the travel restrictions during COVID-19, group interviews were conducted by the Zimbabwe team with students focused on health, food and microbes.^23^ To strengthen the interview data, the participant observation method was used. This aided in structuring and facilitating the assessment of responses and interactions during the interview process and provided further depth and clarity to what the participants were saying.

One researcher taking notes led the group interview while the other researcher followed the observation schema, capturing the group’s non-verbal communication. One hundred and twenty students, aged 16–18 years (Forms 5 and 6), 40/60 gender split, were conveniently sampled from six upper secondary schools in the Midland Region and Gweru District. The interview groups of 20 students were from three school clusters: two private schools/mission schools in periurban areas; and four government schools, two in urban and two in high-density areas. Interviews were conducted in English with each 60 min group interview combining methods to structure data collection and account for unexpected outcomes with students as knowledge co-creators of contextual health. Interview and observation notes, and audio recordings were transcribed by the researchers to add further depth and improved accuracy to the transcription process.^23^ Leading data analysis, the Swedish team worked together with the Zimbabwean team, guided by the conceptual framework and the analytical approach.

### Analytical approach

The study used inductive content analysis,^25^ offering theoretical flexibility in analysing qualitative data. We moved through several analytical steps in analysing and reporting categories and subcategories. (i) We read through the transcriptions to create an overview of the data, followed by an in-depth reading. (ii) We structured the data by identifying key sentences and groups of sentences carrying meaning in relation to the research objective. (iii) In the next step, these key sentences were coalesced to create more succinct meanings. (iv) From key sentences, codes were created as crucial meaningful words or phrases as a basis for analytical categories and subcategories. In the analysis, two categories were identified, each with three subcategories (Table 1*)*.

**Table 1.** Identified analytical categories and sub-categories

Categories	Subcategories
Microbial relationships	Nutrition recycling and decomposition
	Human bodily functions
	Disease-causing bacteria in humans and animals
Encountering microbes	Health-promoting encounters in food
	Risk encounters in public places
	Risk encounters in food

A conceptual framework was developed to add analytical depth, which understands learning as motivated by ‘wicked issues’,^26–28^ beyond anyone’s control, necessitating the creation of new knowledge.^29–32^ Mediating artefacts as material objects that acquire specific meanings and significance through social and cultural mediating experiences and the contextual knowledge of those involved in practice become a way of scaffolding these generative learning processes. A fish is an artefact, i.e. a material object that acquires different meanings for the students if encountered in the practice of eating dinner or biology class dissection.^33^

The conceptual framework was the result of interdisciplinary discussions, including all researchers, aimed at promoting mutual respect and cooperation between the two research teams. Discussions were focused on the identification of shared conceptualizations relevant to the Global South setting of the study.

The framework views health as holistic, created in encounters with social structures as part of lived experiences.^34,35^ This understanding is further strengthened through health literacy, highlighting the need for competence by developing understanding, critical evaluation of health information and ability to take action.^36,37^ According to Van der Heide *et al*.,^38^ health literacy enables exploration of the relationship between education and health, bridging the ‘implementation gap’ between health knowledge and health-promoting practices. Through the capabilities approach,^27,39,40^ the framework comes to consider how commercial, social and political determinants impact students’ capability for developing health literacy.^41–43^ Throughout the analysis, we operationalize the theoretical frame, including health literacy capabilities and mediating artefacts and experiences.^41,42,44^

### Ethics

Before conducting group interviews, permission was granted by the Zimbabwean Ministry of Primary and Secondary Education for conducting the study with students under 18 years old. Information sheets about the aims, process and handling of research results were distributed, along with consent forms, to research participants at preliminary visits. At subsequent visits, opportunities were given to raise questions, and the consent forms, signed by both parents or guardians and the students themselves, were collected. Throughout the group interviews, participants were provided further opportunities to raise questions. The study follows the global code of conduct for research in resource-poor settings.^24^ During data collection, participant codes were used and no participant names were recorded.

## Results

This section presents the results of the group interviews supported by quotes from the students. Two categories are identified, microbial relationships and microbial encounters, each with three subcategories (Table 1). Categories are named after a focus in the interviews with subcategories reflecting specific articulations of the category.

### Microbial relationships

The microbial relationship category focuses on how students related to the microbial world. Three subcategories were identified around the relationship to microbes: (i) nutrition recycling and decomposition; (ii) human bodily functions; and (iii) disease-causing bacteria in humans and animals.

#### Nutrition recycling and decomposition

Recurring in students’ discussions were microbes as building life, with the focus of the subcategory being how humans, animals and crops benefit from microbial nutrition recycling. The two quotes below illustrate students’ awareness of microbes as recyclers of primary elements in living systems, with students highlighting microbial decomposition contributing to soil structure and cycles of nature.

*‘Photosynthetic bacteria living in lakes, ponds and shallow oceans produce the oxygen we breathe.’*

*‘Microbes are beneficial because during the process of decomposition, chemicals such as carbon, nitrogen and phosphorus are released that can be used to build new plants and animals.’*

The key takeaway is how microbes form beneficial relationships with all life: humans, animals and plants.

#### Human bodily functions

Students also highlighted how our ability to absorb nutrition largely relies on microbes. This reflects a recurring topic of discussion among the students that microbes were presented as having positive impacts on our microbial gut flora for bodily functions, as illustrated in the quote below emphasizing a positive microbial relationship as:

‘*Good bacteria in our mouth prevent fungal growth so microbes are crucial.’*

Crucially, with the discussion turning to food, the notions of microbial encounters began to broaden and came to include students’ further positive relationships with microbes. As highlighted by the two quotes below:

*‘Microflora helps in the production of vitamins, stimulation of cell maturation and stimulation of the immune system.’*

*‘The natural flora of microbes in the human gut is essential as part of the digestive system in assisting in breaking down carbohydrates, destroying harmful bacteria, producing hormones and for fat storage.’*

Microbes came to be described by the students, as illustrated by the two quotes below, as sharing spaces with humans on the skin, in the gut and as part of mucous membranes.

*‘Microbes are found everywhere, for example on our skin, noses, hair etc.’*

*‘I know that microbes are in the gut though they are not harmful.’*

The key takeaway from this subcategory is the health-promoting relationships we develop with microbes in our bodies and on our skin, which centres on digestive processes that power our daily life.

#### Disease-causing bacteria in humans and animals

According to the students, microbes appeared in multiple subjects, reflective of their role along the entire food supply chain. This includes how food from animals can become infected during farm production and storage. As seen in the quote below, the students were aware of microbes as the causes of diseases such as cholera, typhoid and runny stomach. As such, students were aware of several harmful microbes associated with health complications for humans.

*‘I learned that common microbes in our country are Mycobacterium tuberculosis, Streptococcus, Salmonella and Chlamydophila pneumoniae.’*

Microbes were thus identified as harmful to humans, characterized by the illnesses they caused, leading to hospitalization and potential death. Furthermore, the quote below showcases how students also extended microbial risks of harmful bacteria to livestock.

*‘We learned how food-producing animals, namely cattle, chickens, pigs and turkeys, are the major reservoirs for many microbes such as Campylobacter*.’

The key takeaway from this subcategory includes the challenges of living with and developing relationships with microbes when co-existence turns into infection, disease and death for humans as well as animals.

As seen throughout these three subcategories of the category *microbial relationships*, students’ discussions included both positive and negative characteristics and while often emphasizing human health also included the planetary benefit of decomposition and the effect on the life of farming animals. Importantly, when students talked about these relationships, they were specific occurrences, which is the focus of the next category, *microbial encounters*.

### Microbial encounters

The focus of the microbial encounters category is how students talked about spaces and places in which they meet microbes and thus interact with the microbial world. Three subcategories were identified from students talking about their meeting with microbes and the impact of these meetings both positively and negatively: (i) health-promoting encounters through food; (ii) risk encounters in public places; and (iii) risk encounters through food.

#### Health-promoting encounters through food

As illustrated in the two quotes below, students noted that microbes had a role in our everyday life practices of making and processing foods, exemplified by how lactobacilli bacteria are used in making yoghurt and how yeast is used in the rising and bread-baking processes.

‘*I became aware that not all microbes are harmful; some microbes like lactobacilli bacteria are important during yoghurt making.’*

*‘Biology made me aware of how microbes work in our bodies, how good and bad they are to us and how they are used in the food industry.’*

Furthermore, students discussed how beneficial encounters with microbes extended to animals as seen in the quote below.

‘*In agriculture, we learnt about the importance of microbes when we discussed animal nutrition.’*

The key takeaway from this subcategory is how food becomes an area in which humans, as well as animals, have beneficial encounters with the microbial world.

#### Risk encounters in public places

The focus of the subcategory of high-risk encounters in public places is how, when speaking about encountering microbes, students saw these encounters occurring everywhere, in diverse situations and places. As seen in the two quotes below, students named places of high-risk encounters with microbes as public places, toilets and public transport, and when cleaning areas and surfaces and handshakes.

*‘We encounter microbes in various places like toilets; they will be in the air, door handles, handwash bowels, even on toilet papers, which will be in the toilets.’*

*‘When one boards a public transport one has no control over the others so some people might not put on their masks and windows closed, after coughing one wipes using bare hands and those same hands will open the door and the next person opens the door.’*

In response to these high-risk encounters, which extended to bad hygiene practices, eating habits and food handling that could enable the transmission of microbes into humans, students assumed a risk terminology in their discussions aimed at the separation of bodies from a risky microbial world.

Microbes were in these discussions, not part of creating health, but rather health was created despite the presence of microbes. Microbes became a source of risk to the students’ bodily health. As such, students were talking about encountering microbes operationalizing a biomedical approach to health; health was in here, in the bodies of the students, and microbes were out there to be avoided, kept out and addressed through hand washing. As illustrated by the two quotes below, students proposed disinfecting and sanitizing surfaces they come into contact with in the home, classrooms, public spaces and open surfaces, as well as themselves using hydrogen peroxide, bleach, vinegar, lemon and essential tea tree oils as their primary practices of co-existing with microbes.

‘*When at home, bleach should be used to wash dishes and sanitize our hands all the time.’*

*‘Tea tree oils can be used for applying on hands after every hand wash.’*

To this end, students were talking about preventive actions to avoid microbial encounters and thus separate themselves from the microbial world through what was described as good hygiene practices. The key takeaway from this subcategory is how encounters of concern for human health often occur in public places and the necessity of preventive practices to protect against unnecessary exposure to harmful microbes.

#### Risk encounters through food

The focus of the subcategory highlighted the microbial risk of food from infected animals, contamination through food processing, preparation and transit as well as the consumption of contaminated food. These concerns, as highlighted by the quote below, extended to the risk posed by poor hygiene practices at food vendors, representing entry points for disease-causing microbes into the food system and subsequent threats to food safety and security.

*‘Ingestion of contaminated food or water, direct contact with infected animals or consumption of food from infected animals can also be a way of transferring microbes from animals to humans.’*

Furthermore, as seen in the quote below, the notion of risks of food-related microbial encounters also came to include how bacteria cause diseases in livestock and animals important to human health in Zimbabwe, such as cattle, poultry, goats and sheep, resulting in less productive animals or even animal death, thus threatening food security among the Zimbabwean population.

*‘Due to high demand for meat in Zimbabwe, people may risk selling contaminated animal products, slaughter a sick cow and sell the meat.’*

Responding to these health concerns, students discussed preventive measures on their part as illustrated by the quote below.

‘*Another good hygiene practice is washing hands before and after eating.’*

A further quote shows how personal hygiene and disinfection also covered the handling of food in the kitchen as part of the students’ descriptions.

‘*When you open any tinned food put it in a closed container to avoid contamination. Cover food with a food cover to avoid flies getting on the food.’*

Participants furthermore highlighted how risk encounters with contaminated food were connected to questions of antibiotic use as knowledge of antibiotics would strengthen people’s agency in managing their own health.

*‘Knowledge on antibiotics will help us not to eat animal meat which we buy in streets and not finishing courses when prescribed antibiotics by doctors.’*

*‘Due to high demand of meat in Zimbabwe, people may risk selling contaminated animal products’*

Expressing the perception of contaminated animal products as a reason for needing antibiotics, students went on to note that infections acquired in risk encounters through food could exacerbate AMR due to poor adherence to the prescribed course of antibiotics, as seen in the quotes below:

‘*When people take antibiotics at home they are told by doctors to complete the course, but they throw away the antibiotics or just stop taking them once they feel they are ok.’*

*‘We have a tendency of not finishing the course and if a family member gets sick we just give the remaining medication to the sick which does not make up a course and that will cause resistance.’*

The students indicated that co-existence was possible when exercising good personal hygiene: washing your body every day, washing hands with soap after visiting the toilet, brushing your teeth twice a day, covering your mouth and nose when sneezing or coughing, and cutting or cleaning fingernails.

The key takeaway from this subcategory is how food all the way from farm to table encompasses potential health risks for humans while at the same time emphasizing how preventive and protective measures are literally in the hands of everyone.

As seen throughout these three subcategories of the category *microbial encounters*, students discuss microbial encounters as they occur throughout their everyday lives, from the risks posed by moving through public places to both the benefits and the potentially harmful impact of our ongoing food practices.

## Discussion

**Figure 1.**
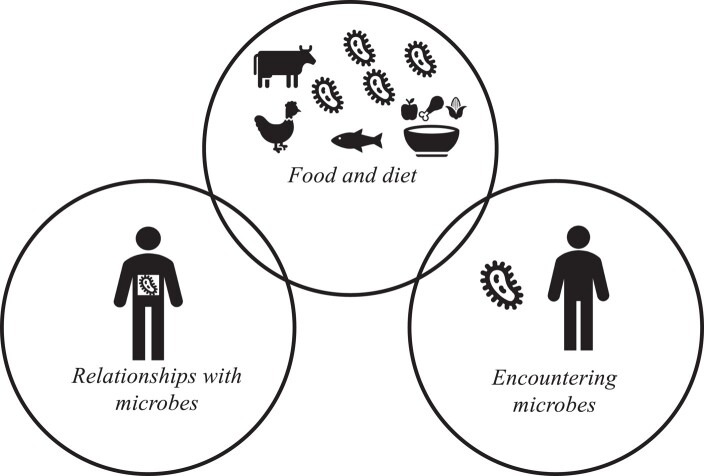
Microbial relationships and encounters through food and diet.

In the results, students were simultaneously aware of how relationships and encounters with the microbial world involve benefits and risks. In line with Boorse,^20^ students expressed pathogenic perspectives on how microbes pose risks for infection and disease. Students’ discussions emphasized the risks posed by contaminated foods such as street meat for infection and subsequent needs for courses of antibiotics. In light of the social and cultural context in which these antibiotics are taken, students highlighted the obstacles for completing a course due to patients feeling better and the social pressures of sharing valuable medication with friends and family. To this end, contaminated food was linked in the discussions to the development of AMR. Participants furthermore highlighted how risk encounters with contaminated food were connected to questions of antibiotic use, as knowledge of antibiotics would strengthen people’s agency in managing their own health. Expressing the perception of contaminated animal products as a reason for needing antibiotics, students went on to note that infections acquired in risk encounters through food could exacerbate AMR due to poor adherence to the prescribed course of antibiotics. Meanwhile, students also articulated understandings closer to the holistic approach of Ewles and Simnett^7^ when discussing health-related practices and life experiences. Microbes became, in the discussions, a source for creating bodily health in line with Van De Pas^5^ and Zinsstag,^6^ which was accompanied by the introduction of food and eating. As an individual and communal practice, with family or friends, at home or on the move, food and eating became a source for experiential knowledge, as highlighted by Ben *et al.*^21^ and Aslam *et al.*,^22^ of the effects of microbes in students’ lives, encountering and creating health together with microbes. As argued by Hernando-Amado *et al.*^14^ and Ma *et al*.,^15^ these health-promoting relationships extended to the food supply chain with health as a practice and the negative narrative of biomedical societal health overlapping with the positive narrative of holistic health in the lives of students (see Figure 1).

Furthermore, students’ discussions centred on the microbial flora as essential to our digestive system in breaking down carbohydrates, destroying harmful bacteria, producing hormones and for fat storage. Microbial encounters, through food and diet, went from occurring ‘out there’, as external risks to students’ health, to equally occurring ‘in here’ through health-promoting bodily processes.

**Figure 2.**
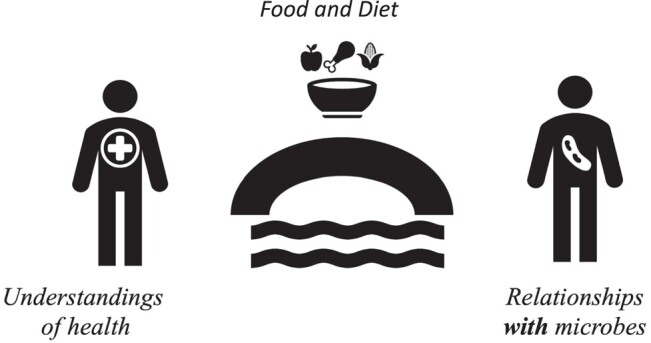
Food and diet connecting understandings of health and the microbial world.

Food emerged as mediating artefacts^44^ connecting students’ understandings of health and the microbial world through dairy and the traditional foods of sadza (see Figure 2). Eating practices become a mediating experience where students see, feel and taste the impact of the microbes, coming to know the microbial world through experiences with food.

Food practices thus included engagements with the microbial world in the context of bodily health and as part of AMR emergence and spread. Students experienced agency in being able to promote their health by engaging in actions, which according to Eleraky *et al.*,^8^ contributes in addressing AMR. In line with Wood’s^2^ and Maccaro’s^3^ move away from the war metaphor, our findings showcase how students are able to develop relationships with the microbial world that extend beyond the elimination of health risk to include holistic notions of health, in line with the One Health of McEwen *et al*.^45,46^ As noted by Pithara,^41^ Ruger^42^ and Van der Heide *et al.*,^38^ our findings show students creating critical conceptual understandings of the health of themselves and their community.

This paper makes a contribution by highlighting how it is necessary to consider the contextual motivations of participants and engage with these motivations and reasons when engaging with information, communication and education as means of behaviour change in improving understanding and action on AMR.^4,9^ Leveraging the insights gained from the interviews, it’s possible to develop a more targeted and effective strategy for facilitating behaviour change. This entails identifying behavioural patterns connected to AMR and, secondly, understanding the motivations behind these behaviours, exploring together the meanings given to behaviours by participants. In light of this deeper understanding of existing AMR behaviours, an educational process can support participants in articulating behavioural change goals and facilitating the process of behaviour change, ensuring that individuals have access to the tools they need. Through the use of monitoring and evaluation coupled with incentives for participants, the progression of behaviour change could be monitored and necessary adjustments to the approach could be made. Educational processes thus become crucial in the move towards effective health communication, especially for ‘wicked’ sustainable health challenges such as AMR that require novel and contextual solutions.^26,27,47^ Responding to the research objective to study experiences, understanding and practices of Zimbabwean students regarding health, food and microbes, the paper illustrates the crucial role of food and eating as mediating artefacts and experiences. Food and eating as mediating artefacts and experiences can thus support students’ agency in becoming the storytellers of their relationships with the microbial world, enabling students to engage with what cannot be seen and inform how AMR education is articulated in practice.
